# The role of artificial intelligence in enhancing sports education and public health in higher education: innovations in teaching models, evaluation systems, and personalized training

**DOI:** 10.3389/fpubh.2025.1554911

**Published:** 2025-04-30

**Authors:** Yan Gao

**Affiliations:** Capital University of Physical Education and Sports, Institute of Artificial Intelligence in Sports, Beijing, China

**Keywords:** artificial intelligence, colleges and universities, public sports, teaching reform, public health

## Abstract

With the rapid development of artificial intelligence (AI) technology, particularly in the field of physical education in higher education institutions, the application of AI has shown significant potential. AI not only offers innovative teaching models and evaluation systems for physical education, but also enhances teaching efficiency, enables personalized instruction, and improves students' athletic performance. In the context of public health, AI's role becomes even more crucial, as it assists in developing scientific exercise plans through precise motion data analysis, thereby promoting both physical and mental health. Furthermore, AI technology can drive innovation in the content and methods of public physical education teaching, providing robust support for high-quality sports education. Studies indicate that AI has optimized the physical education process, spurred the innovation of curriculum content, and facilitated the transformation of teaching models, injecting new momentum into the sustainable development of physical education in universities and the achievement of public health goals.

## 1 Introduction

With the rapid advancement of artificial intelligence (AI) technology, its application in higher education physical education (PE) has transitioned from experimentation to practical implementation. Research suggests that AI-driven reforms are transforming traditional PE methods into intelligent, data-driven instructional models (1). By integrating real-time data analysis, AI enables personalized teaching adjustments based on students' learning progress and athletic abilities, significantly enhancing engagement and performance. A survey of 200 students revealed that AI-assisted teaching increased student motivation by ~30% (1). Beyond skill improvement, this approach also contributes to long-term physical health and helps mitigate public health risks such as obesity (2).

AI-based motion analysis further enhances teaching quality by enabling precise, data-driven instructional strategies. Motion recognition systems track students' movements in real-time, providing accurate feedback and refining technique execution. Consequently, AI-driven intelligent curriculum reform has gained widespread adoption, making PE instruction more personalized and adaptable (3, 4). For instance, an AI-integrated PE reform experiment showed that 80% of 150 participating students reported better mastery of athletic skills through AI-assisted courses, leading to sustained interest in physical activity (3). Furthermore, AI has introduced advanced assessment methods that enhance accuracy and personalization. AI systems analyze students' athletic data to develop individualized learning paths, significantly improving performance, particularly in coordination and endurance (5–7). These systems continuously monitor movement patterns, offering real-time feedback and adjusting training accordingly, thereby optimizing skill development and facilitating early detection of potential health risks (8).

From a broader public health perspective, AI-driven interventions support early identification of health issues and personalized exercise plans, aligning PE with public health objectives (8). Additionally, AI necessitates upskilling among educators. A survey of 500 PE teachers indicated that 60% lacked adequate AI training, hindering effective technology integration. To address this, universities are introducing AI training programs to enhance teachers' proficiency in data analytics and AI-assisted instruction (8, 9).

Additionally, AI holds significant potential in preventing sports injuries by integrating physiological data (e.g., electromyography, heart rate variability) and employing machine learning to predict injury risks (10). AI-driven biomechanical modeling can identify movement inefficiencies, optimize training, and reduce fatigue-induced errors (11). Wearable AI systems provide real-time feedback, preventing overtraining and enhancing recovery strategies (10). Future AI-driven rehabilitation programs will further improve performance and injury prevention.

This paper explores the role of AI in higher education PE, with a focus on enhancing athletic performance, promoting health management, and achieving public health goals. The findings provide valuable insights for the future development of university sports programs.

## 2 AI's role in public physical education reform

AI is transforming higher education PE by introducing personalized instruction, intelligent assessment, and teacher development strategies. Unlike traditional “one-size-fits-all” methods, AI-driven systems create customized learning experiences based on individual physiological and performance data, thereby optimizing training intensity and minimizing injury risk (5–7).

AI enhances teaching effectiveness through real-time performance tracking and feedback. By leveraging data analytics, teachers can dynamically adjust lesson plans to align with students' progress, shifting from conventional “one-to-many” instruction to an interactive, student-centered approach (1, 12–14). Immersive technologies such as Virtual Reality (VR) and Augmented Reality (AR) further boost engagement, making physical education more effective and enjoyable (15).

Furthermore, AI significantly improves injury prevention in sports training. By analyzing movement biomechanics and identifying inefficient motion patterns, AI can help athletes refine their techniques and reduce the likelihood of overuse injuries (16–18). Research indicates that AI-based motion recognition systems can predict injury risk with 85% accuracy by assessing an athlete's movement deviations and muscular imbalances (17). Wearable AI technologies further enhance real-time monitoring, allowing for immediate feedback and personalized adjustments in training loads to mitigate injury risks (19). AI-driven rehabilitation programs also accelerate post-injury recovery by customizing exercises based on real-time physiological responses, thereby improving rehabilitation outcomes and minimizing re-injury rates.

Additionally, AI facilitates professional development for educators. Since effective AI integration requires technical proficiency, teacher training programs are increasingly incorporating AI literacy modules (9, 10). AI-driven platforms also promote equitable access to quality PE resources, benefiting students in under-resourced areas (20, 21). By enabling data-informed decision-making, AI helps ensure that PE interventions effectively enhance student fitness and align with public health objectives (9, 10).

## 3 Pathways for AI to enhance higher education PE

To achieve high-quality development in PE, institutions must invest in AI-driven innovations, optimize resource allocation, and adopt technology-enhanced training methods. AI improves efficiency in resource utilization by analyzing student engagement metrics and optimizing facility usage, leading to a 20–30% increase in effectiveness (22, 23). Empirical data indicates that AI-driven optimizations significantly boost student participation and attendance in PE programs. For instance, a university implementing AI-assisted training reported a 25% rise in student participation and a 15% increase in class attendance (24–26). AI dynamically tailors course content based on real-time feedback, ensuring personalized and engaging learning experiences.

Beyond PE, AI also plays a crucial role in public health management. Large-scale data analytics enable policymakers to develop targeted health promotion strategies, addressing prevalent issues such as chronic disease and sedentary lifestyles (27–30). Moreover, AI-powered VR and AR applications provide inclusive exercise opportunities for individuals facing spatial or physical constraints, expanding access to fitness resources (31–34). AI also enhances teaching quality through real-time feedback mechanisms. By continuously tracking students' exercise metrics, AI helps educators refine instructional methods, thereby improving student outcomes (35). Additionally, AI supports health monitoring by identifying potential risks and providing personalized recommendations, reinforcing the role of PE in long-term wellbeing (13, 36).

AI's role in performance enhancement extends to personalized athletic training. AI-based systems analyze an athlete's biomechanics and physiological data to create customized training regimens that optimize performance while minimizing fatigue (37). Research has shown that AI-assisted training improves strength, endurance, and coordination by up to 20% compared to traditional training programs (38). Furthermore, AI-driven predictive analytics can identify potential performance plateaus, allowing for timely modifications in training plans to sustain continuous improvement (35).

In facility management, AI-driven smart sports venues optimize environmental conditions to enhance exercise experiences. Automated adjustments to temperature, humidity, and lighting contribute to improved performance and injury prevention (39, 40). Smart booking systems further streamline access to sports facilities, increasing student participation in physical activities (41).

Although artificial intelligence demonstrates significant potential in higher education physical education (PE), this study has certain limitations. Firstly, the accuracy and generalizability of current AI systems in motion analysis are still constrained; individual differences and diverse sporting contexts may lead to biases in data interpretation. Secondly, some findings rely on survey data and case studies, which may lack sufficient sample size and experimental control. Additionally, the varying levels of AI literacy among educators could impede the effective integration of AI technologies into teaching practices. Future research should focus on strengthening empirical validation, expanding participant samples, and enhancing the usability and adaptability of AI-assisted instructional tools (Table 1).

**Table 1:** Applications of artificial intelligence in higher education physical education (PE).

**Module**	**Key content**	**Specific outcomes**	**References**
Teaching reform	AI supports personalized instruction and real-time, data-driven teaching adjustments	Student motivation increased by ~30%; 80% of students showed improved skill mastery	(1, 3)
Motor skill optimization	Use of motion recognition and feedback technology to enhance movement execution	Improved accuracy and comprehensive technical proficiency	(3, 5, 6)
Health promotion	Development of individualized health plans based on data analysis	Helps prevent obesity and enhances coordination and endurance	(2, 7, 8)
Injury prevention	AI integrates physiological data (e.g., EMG, HRV) to predict injury risks	Achieved 85% injury prediction accuracy; reduces risks from training overload	(10, 11, 19)
Intelligent assessment	AI-driven evaluation systems provide dynamic performance feedback	Enhances assessment accuracy and personalization	(5, 7)
Teacher development	Promotes AI literacy and technical skills training for educators	60% of PE teachers lack adequate AI proficiency, requiring systematic training	(8, 9)
Resource Optimization and Management	AI improves resource allocation for venues, equipment, and curriculum	Student participation increased by 25%; class attendance increased by 15%	(22, 24)
Virtual/augmented reality	Use of VR/AR enhances engagement and immersive learning experiences	Increases teaching effectiveness and overcomes spatial limitations	(15, 31, 32)
Public health support	Big data-driven strategies for group health interventions	Enables customized public health programs and chronic disease prevention	(31, 34, 36)
Future development	AI-driven intelligent rehabilitation and fatigue monitoring systems	Improves rehabilitation outcomes and reduces reinjury risk	(10, 11)

## 4 Conclusion

The integration of AI in higher education PE is revolutionizing instructional models, improving teaching quality, and advancing public health initiatives. AI-driven data analysis and intelligent assessments facilitate personalized training programs, optimizing student performance while preventing injuries. The adoption of immersive technologies such as VR and AR further enhances engagement and participation, breaking traditional barriers to sports education.

AI is also reshaping teacher training, resource allocation, and public health strategies, promoting equitable access to quality PE. As AI continues to evolve, its applications in sports education will expand, fostering smarter, more effective training methodologies and supporting national fitness initiatives. By leveraging AI's capabilities, universities can drive innovation in PE, ultimately contributing to a healthier society.

## Data Availability

The original contributions presented in the study are included in the article/supplementary material, further inquiries can be directed to the corresponding author.
